# The Crucial Role of Physiotherapy in the Postoperative Case of Temporomandibular Joint Ankylosis for Restoration and Normalization of Functions in a 23-Year-Old Patient

**DOI:** 10.7759/cureus.62291

**Published:** 2024-06-13

**Authors:** Nikita Bhusari, Pooja Dhage, Prasad P Dhage

**Affiliations:** 1 Musculoskeletal Physiotherapy, Ravi Nair Physiotherapy College, Datta Meghe Institute of Medical Sciences, Wardha, IND

**Keywords:** mandible mobility, facial asymmetry, trauma, children, temporomandibular joint ankylosis

## Abstract

Ankylosis of the temporomandibular joint (TMJ) is a pathological condition of the joint. The disease manifests as a limitation to total failure of movement of the TMJ, usually following trauma or surgery or due to local infection. The condition may result in difficulty masticating, speaking, structure of the mouth, face, or jaw, and maintaining oral hygiene to a significant degree. A computed tomography (CT) scan is the best method of evaluating the bony anatomy of the TMJ. The present report shows the surgical correction of the TMJ ankylosis. A 23-year-old female attended the hospital, showing severe mouth opening limitation (9 mm). On investigations, left TMJ ankylosis was diagnosed. The surgical approach consisted of distraction osteogenesis of the left side, followed by vigorous physiotherapy. In patients with TMJ ankylosis, restoration of normal function and jaw movement is difficult. This case report highlights the importance of physiotherapy as an emerging adjuvant therapy in the treatment of TMJ ankylosis. There have also been several treatment methods used to improve the patient's self-esteem and confidence, including speech therapy and psychological counseling.

## Introduction

The temporomandibular joint (TMJ) is a complex skeletal structure, but it is important for the normal functioning of the jaw. Also, it is considered the most active functioning joint of the body. It consists of two joints with bilateral synovial articulations that connect the mandibular bone to the skull’s temporal bone below [1]. Ankylosis is characterized by the stiffness of a joint due to abnormal adhesions and rigidity of the bones. This occurs when the movement of the condyle is restricted, resulting in a partial or complete inability to open the mouth [1]. One of the most devastating and difficult disorders to diagnose, TMJ ankylosis affects many aspects of a patient's life, including chewing, speaking, and aesthetic aspects. Early TMJ ankylosis in children can cause facial asymmetry, growth disruptions, eating and breathing problems, as well as major respiratory problems when sleeping [2]. Children with unilateral TMJ ankylosis experience facial asymmetry as a result of the chin deviation to the affected side. It is important to perform a detailed preoperative radiographic assessment of the type and extent of the ankylosis to determine an effective treatment [3].

Each patient with ankylosis of TMJ requires a history, physical examination, and radiographic examination to reach a definite diagnosis, determine the severity, determine whether neighboring tissues are involved, and eventually plan the course of treatment [1]. Due to the low prevalence of TMJ ankylosis, there is an absence of developed recommendations concerning physiotherapeutic procedures in children and adults [4]. Several key management principles are involved in this procedure, including adequate removal of the ankylotic mass, costochondral grafting, and postoperative physical therapy [5]. Usually, a dentist will notice an increasing limitation of jaw opening as the first indication of a significant problem. It is uncommon to experience pain. To prevent the worst consequences of this condition, early diagnosis and treatment are imperative [6]. The management of TMJ ankylosis is challenging due to the high incidence of recurrence and the absence of a published consensus [7]. Physiotherapy aims to manage pain, reduce swelling, and maintain jaw mobility through exercises that improve jaw mobility, muscle strength, and coordination.

## Case presentation

Patient information

**Figure 1:**
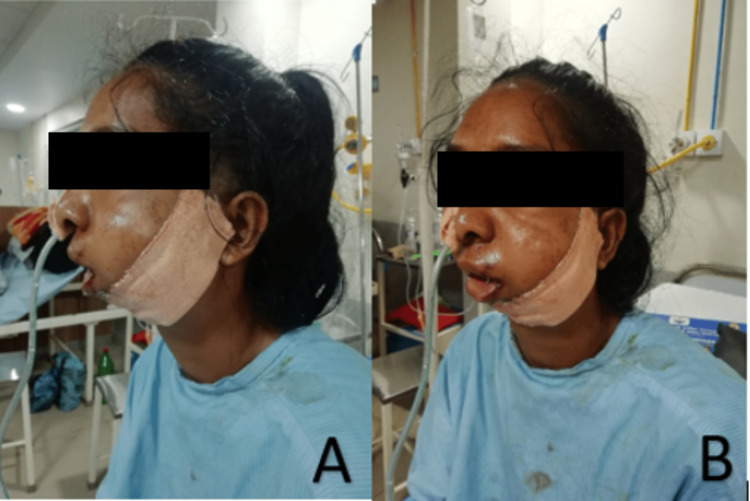
(A) Side view showing facial fullness. (B) Oblique view showing facial fullness.

**Figure 2:**
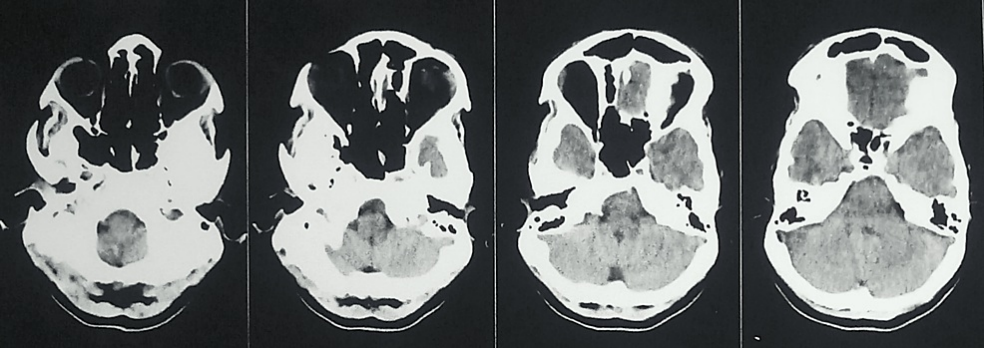
CT scan showing deviation of the mandible to the left.

**Figure 3:**
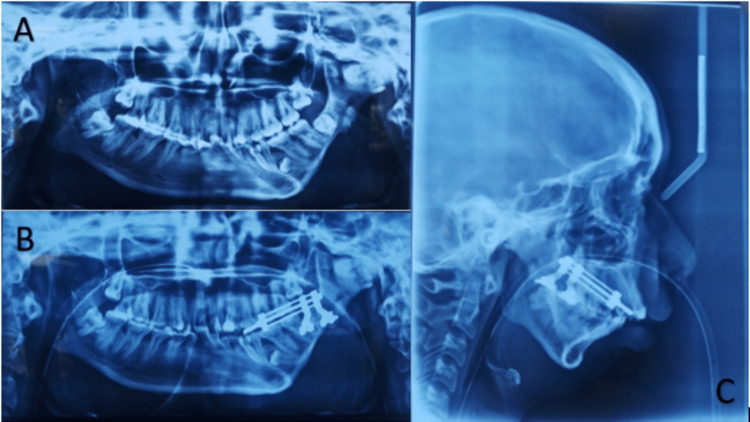
(A) Preoperative X-ray. (B) Postoperative X-ray. (C) Lateral view X-ray

A 23-year-old girl reported the inability to open her mouth since birth. The patient was born after completing a gestational period of nine months by vaginovertex delivery. The milestones were achieved on time. A gradual reduction in mouth opening was observed, resulting in her inability to eat properly. There was asymmetry in the face, resulting in chin deviation to the left. On the left side, there was facial fullness, whereas on the right side, there was facial flatness, as shown in Figure 1. A convex facial profile was observed. It was almost impossible for the patient to open his mouth. In the radiographic examination, computed tomography (CT) was performed, revealing the presence of radiolucent lines inside the fusion area but no recognizable condyle and fossa to be found, as well as a widening of the mandibular condyle and temporal aspect of the joint. Retrognathia was also present, along with deviation of the mandible to the left. Bilateral stylohyoid ligaments appear calcified, as shown in Figure 2. Based on these findings, a diagnosis of unilateral grade III bony ankylosis of TMJ on the left side was confirmed. The surgical approach consisted of distraction osteogenesis of the left side, as shown in Figure 3.

The patient was evaluated using manual muscle testing and the cervical joint's range of motion (Table 1).

**Table 1:** Assessment

Variable	Movement	Postoperative day 1
Range of motion - cervical joint	Flexion	0-10°
	Extension	0-10°
	Lateral flexion	0-15°
	Shoulder flexion	0-120°
	Shoulder Abduction	0-110°
	Medial rotation	0-90°
	Lateral rotators	0-90°
Manual muscle testing	Cervical flexors	3-/5
	Cervical extensors	3/5
	Side flexors	3/5
	Shoulder flexors	4/5
	Shoulder extensors	4/5
	Abductors	4/5
	Adductors	4/5
	Medial rotators	4/5
	Lateral rotators	4/5

Pain Assessment

The grade of tenderness was 2. On the Numerical Pain Rating Scale, the score was 6/10 on movement and 5/10 on rest.

Physiotherapy management

The patient was treated according to the physiotherapy protocol listed in Table 2.

**Table 2:** Intervention

Sr. no.	Goal	Management	Regime
1	Pain management	Cryotherapy around the suture site	15 min
2	To increase mouth opening and mouth deviation exercises	Mouth opening and closing exercise	10 reps × 1 set
3	To maintain the active range of motion of the cervical joint	Cervical movements for a range of motion exercises	10 reps × 1 set
4	To prevent contracture	Upper limb and lower limb mobility exercises and mobilization	10 reps × 1 set
5	To maintain lung volume	Breathing exercises	10 reps × 1 set

Outcome measures (Table 3), such as the Numerical Pain Rating Scale (NPRS), Modified Mallampati Scale, and Barthel Index, were used to evaluate the patient.

**Table 3:** Outcome measures

Outcome measures	Preoperative	Postoperative
Numerical Pain Rating Scale (NPRS)	6/10	4/10
Modified Mallampati Scale	Class IV	Class II
Jaw Functional Limitation Scale-20 (JFLS-20)	135/200	105/200

## Discussion

TMJ ankylosis is a significant and disabling condition that can impair facial development, chewing, swallowing, digestion, speech, appearance, and oral hygiene. In addition to causing aesthetic deformities, it can also have functional consequences. Ankylosing spondylitis, rheumatoid arthritis, and psoriasis are all systemic causes of TMJ ankylosis (6). Mouth opening restriction, facial asymmetry, mandibular micrognathia, and class II posterior cross-bite malocclusion are clinical features of TMJ ankylosis in children (unilateral ankylosis)/anterior open bite (bilateral ankylosis) [8].

Ankylosis types and classification were based on Sawhney criteria (Sawhney, 1986): Type I: There is a condylar head present, but it is deformed. Movement is impossible due to fibrous adhesions. Type II: Fusion of misshaped head and articular surface, mainly located on either the anterior or posterior edge of the articular surface. TMJ's medially located pole was not damaged. Type III: Bony block that connects the ascending ramus of the mandible with the zygomatic arch. Medially atrophic and dislocated fragments of the former head of the condyle are still to be found. The upper articular surface and, in rare cases, the articular disc were intact medially. Type IV: Regular anatomy of TMJ totally destroyed by an expanded bony block between the ramus and skull base.

In this case, distraction osteogenesis was performed which is used to lengthen the jawbone, correct deformities, and resolve functional problems. A mandibular distraction osteogenesis procedure generally results in significant improvements in both function and aesthetics. As a result of better jaw alignment and a larger oral cavity, this procedure also improves articulation and clarity of speech.

Preventing postoperative adhesions and re-ankylosis, early postoperative exercises, appropriate physiotherapy, and close patient follow-up are as important as radical and sufficient resection of the ankylosed bone [9]. The treatment of TMJ ankylosis should be initiated as soon as possible [10]. A rigorous physiotherapy program must be implemented to improve mouth opening further and primarily to prevent the reoccurrence of ankylosis.

The study done in 2024 highlighted that bony ankylosis is prevalent in patients with TMJ ankylosis, highlighting its adverse effects. Physiotherapy, however, is essential for optimizing postoperative outcomes and minimizing adverse events such as re-ankylosis. For optimal results, practitioners and healthcare professionals should monitor postoperative recovery and adhere strictly to physiotherapy protocols [11].

## Conclusions

A multidisciplinary approach and team effort are beneficial for treating TMJ ankylosis. Physiotherapy should be initiated early to maximize muscular movements and reset the maximum opening of the mouth, as patients should be closely monitored until the finalization of mandibular development is complete to determine whether any changes need to be made. Physiotherapy interventions have a profound effect on the clinical symptoms of the condition, as well as on the functional level of the patient, demonstrating physiotherapy's effectiveness as adjuvant therapy. In this clinical case, the type of postoperative management can affect the efficacy of surgical procedures. A physiotherapy program initiated immediately following the procedure for releasing the ankylosis was shown to improve the patient's outcome with regard to mandible mobility.
